# Safety and Efficacy of Excimer Laser Powered Lead Extractions in Obese Patients: A GALLERY Subgroup Analysis

**DOI:** 10.3390/jcm12124096

**Published:** 2023-06-16

**Authors:** Niklas Schenker, Da-Un Chung, Heiko Burger, Lukas Kaiser, Brigitte Osswald, Volker Bärsch, Herbert Nägele, Michael Knaut, Hermann Reichenspurner, Nele Gessler, Stephan Willems, Christian Butter, Simon Pecha, Samer Hakmi

**Affiliations:** 1 Department of Cardiology and Critical Care Medicine, Asklepios Klinik St. Georg, 20099 Hamburg, Germany; 2 Department of Cardiology, University Heart & Vascular Center Hamburg at the University Medical Center Hamburg-Eppendorf, 20246 Hamburg, Germany; 3 Department of Cardiac Surgery, Kerckhoff Klinik, 61231 Bad Nauheim, Germany; 4 Division of Electrophysiological Surgery, Johanniter-Hospital Duisburg-Rheinhausen, 47228 Duisburg, Germany; 5 Department of Cardiology, St. Marien Krankenhaus, 57072 Siegen, Germany; 6 Department for Cardiac Insufficiency and Device Therapy, Albertinen-Hospital, 22457 Hamburg, Germany; 7 Department of Cardiac Surgery, University Heart Center Dresden, 01307 Dresden, Germany; 8 Department of Cardiovascular Surgery, University Heart & Vascular Center Hamburg at the University Medical Center Hamburg-Eppendorf, 20246 Hamburg, Germany; 9 Department of Cardiology, Heart Center Brandenburg Bernau, 16321 Bernau, Germany

**Keywords:** obesity, transvenous lead extraction, excimer laser, complications, efficacy

## Abstract

Background: The incidence of cardiac implantable electronic device (CIED)-related complications, as well as the prevalence of obesity, is rising worldwide. Transvenous laser lead extraction (LLE) has grown into a crucial therapeutic option for patients with CIED-related complications but the impact of obesity on LLE is not well understood. Methods and Results: All patients (*n* = 2524) from the GermAn Laser Lead Extraction RegistrY (GALLERY) were stratified into five groups according to their body mass index (BMI, <18.5; 18.5–24.9; 25–29.9; 30–34.9; ≥35 kg/m^2^). Patients with a BMI ≥ 35.0 kg/m^2^ had the highest prevalence of arterial hypertension (84.2%, *p* < 0.001), chronic kidney disease (36.8%, *p* = 0.020) and diabetes mellitus (51.1%, *p* < 0.001). The rates for procedural minor (*p* = 0.684) and major complications (*p* = 0.498), as well as procedural success (*p* = 0.437), procedure-related (*p* = 0.533) and all-cause mortality (*p* = 0.333) were not different between groups. In obese patients (BMI ≥ 30 kg/m^2^), lead age ≥10 years was identified as a predictor of procedural failure (OR: 2.99; 95% CI: 1.06–8.45; *p* = 0.038). Lead age ≥10 years (OR: 3.25; 95% CI: 1,31–8.10; *p* = 0.011) and abandoned leads (OR: 3.08; 95% CI: 1.03–9.22; *p* = 0.044) were predictors of procedural complications, while patient age ≥75 years seemed protective (OR: 0.27; 95% CI: 0.08–0.93; *p* = 0.039). Systemic infection was the only predictor for all-cause mortality (OR: 17.68; 95% CI: 4.03–77.49; *p* < 0.001). Conclusions: LLE in obese patients is as safe and effective as in other weight classes, if performed in experienced high-volume centers. Systemic infection remains the main cause of in-hospital mortality in obese patients.

## 1. Introduction

The prevalence of obesity in developed countries is continuously rising. Approximately one third of the world’s population is currently affected [1,2]. By 2030 almost every second American citizen, as well as more than 30% of Europeans, will be obese [3,4].

As a foundation of the development of metabolic syndrome and obstructive sleep apnea (OSAS), obesity plays a major role as a direct and indirect cardiovascular risk factor [2]. A better understanding of its consequences for medical therapies is paramount.

Besides rising numbers of obesity, age is a demographic factor with a growing impact on medical treatment around the globe. By 2050, 22% of the world’s population will be 60 years and older, which has a big impact on cardiovascular medicine [5].

As a result, numbers of cardiac implantable electronic devices (CIEDs) are high and CIED-related complications leading to the necessity of transvenous lead extraction (TLE) are increasing likewise [6,7]. Transvenous leads are a major risk factor for systemic infection with an indication for a timely extraction. Lead-associated vascular pathology (e.g., occlusion, stenosis), as well as lead dysfunction, add to the list of indications for TLE [7,8].

With a longer lead dwell time in an aging population, the complexity and with it the complication rate of TLE procedures is rising [9]. The application of excimer laser powered extraction tools can be of help in these situations, and has shown good safety and efficacy [10,11,12].

In surgery, obese patients tend to suffer from higher complication rates and longer healing times, although a so-called “obesity paradox” (patients with mild-to-moderate overweight/obesity showed reduced complication rates after abdominal or heart surgeries compared to normal weight patients) has been described for certain settings [13,14,15]. Additionally, several studies also displayed worse outcomes for underweight patients with a low body mass index (BMI) [14,15].

Information about the outcome in obese patients undergoing interventional procedures with smaller access areas (catheter insertion site), and shorter procedural and sedation time is scarce. Recently, a study showed no influence of the BMI on catheter ablation for cardiac arrhythmia [16]. The impact of obesity on lead extraction procedures, especially on excimer laser powered TLE procedures (LLE), is not known, although several trials identified a BMI < 25 kg/m^2^ as an independent predictor of 30-day all-cause mortality after TLE [11,17]. Several risk scores for adverse events after TLE have been proposed recently, but none of them included underweight or overweight in their calculations [18,19].

Therefore, the objective of this GALLERY (GermAn Laser Lead Extraction RegistrY) subgroup analysis is to clarify the safety and efficacy of LLE procedures in obese as well as underweight patients, stratified by BMI.

## 2. Methods

### 2.1. Study Population

Between 2013 and 2017, the GALLERY enrolled 2524 patients with a total number of 6117 leads undergoing laser lead extraction (LLE) from 24 centers across Germany. The current subgroup analysis focused on different weight classes divided by BMI (kg/m^2^) according to the current World Health Organization (WHO) definition with special focus on patients with obesity: all patients with a BMI of <18.5 kg/m^2^ were classified as underweight, patients with a BMI of 18.5–24.9 kg/m^2^ were defined as normal weight and patients with a BMI of 25–29.9 kg/m^2^ were defined as overweight. Additionally, obesity class 1 was defined as a BMI of 30–34.9 kg/m^2^ and patients of obesity class 2 had a BMI of greater than 35 kg/m^2^. The methodology of data collection and design of the main GALLERY study have already been published [8]. This study is fully in accordance with the Declaration of Helsinki and the study protocol was accepted by the ethics committee of the State Medical Board Hamburg (reference number:WF-026/17).

### 2.2. Definitions

The definitions regarding LLE procedure, including all utilized techniques, tools, procedural complications and outcomes, are based on the current HRS and EHRA expert consensus statements [20,21]. Complete lead removal was achieved, if the entirety of the targeted lead material was extracted from endovascular space. Incomplete lead removal was determined, if whole leads or fragments >4 cm thereof were left inside the patient at the end of procedure. Complete procedural success was achieved with complete lead removal in the absence of any permanently debilitating complications or procedure-related death. Clinical procedural success was defined as achievement of the procedural and clinical goals of the LLE, despite smaller retained lead fragments in the absence of any permanently debilitating complications or procedure-related death. Procedural failure was determined, if complete procedural or clinical success could not be achieved, and/or in the event of any permanently debilitating complications or procedure-related death. Major complications occurred in any procedural adverse event, which were either life-threatening, resulted in death or any other permanently debilitating condition. Minor complications occurred in any procedural adverse event, that ensued from a medical or minor procedural intervention without lasting impact on patient’s functional capacity. Procedure-related death occurred in cases of intraprocedural death or any death, that was directly or indirectly associated with a procedural complication. In-hospital mortality was defined as any cardiac or non-cardiac fatality during the hospital stay, regardless of its relation to the procedure. 

### 2.3. Periprocedural Management and LLE Procedure

The utilization of powered extraction tools, such as excimer laser sheaths, became necessary to address endovascular fibrous adhesions of targeted leads, that had prevented safe transvenous lead extraction for such patients in the past. The laser operates with a wavelength of 308 nm with a penetration depth of 0.05 mm and dissolves tissues (e.g., lead adhesions) on contact by evaporation, freeing leads from the vascular wall. Since this technology relies on intracellular water to take effect, severely calcified adhesions are addressed with other tools instead, such as mechanical rotating dilator sheaths [22]. All LLE procedures were performed in a hybrid operating theater with fluoroscopy, and fully primed cardio-pulmonary bypass and perfusionist team on standby. Primary extractor was either a cardiologist with a cardiac surgeon at the table or on standby, or a cardiac surgeon alone. Primary tool for extraction was the excimer laser sheath (SLS^®^ II 40 Hz or GlideLight™ 80 Hz, both PHILIPS Healthcare, Amsterdam, The Netherlands). If necessary, other tools, such as mechanical rotating dilator sheaths and snares, were allowed.

### 2.4. Statistical Analysis

Continuous variables are displayed as mean ± standard deviation (SD) for Gaussian distributions, and median and interquartile range (IQR) for non-Gaussian distributions. Categorial variables are displayed as counts and percentages. For intergroup comparisons of categorical variables, Chi^2^-test or Fisher’s exact test in case of small sample sizes (<5 counts per cell) were used. Intergroup comparisons of continuous variables were conducted by Mann–Whitney U test. Continuous variables between >2 groups were compared using the Kruskal–Wallis test. Univariate and multivariate logistic regression analysis was used to identify independent predictors for all-cause in-hospital mortality, procedural complications and failure. Predictor candidate variables that reached statistical significance in univariate analysis and additional clinically relevant covariates were included in the multivariate analyses. A 2-tailed *p*-value of <0.05 was considered as statistically significant. Statistical analysis was performed using Prism 8 (GraphPad Software, San Diego, CA, USA) and IBM SPSS 25.0 statistics software package (IBM, Armonk, NY, USA).

## 3. Results

### 3.1. Patient Baseline Characteristics

The baseline characteristics of all five defined BMI groups are displayed in Table 1.

Combined, all patients who underwent LLE procedures in the 24 participating centers across Germany had a mean BMI of 27.1 ± 4.6 kg/m^2^. The majority of patients (*n* = 1176, 46.6%) were overweight (BMI 25.0–29.9 kg/m^2^). A BMI of 30.0–34.9 kg/m^2^ (obesity class 1) was present in 422 patients (16.7%), whereas obesity class 2 (BMI ≥ 35 kg/m^2^) consisted of 133 patients (5.3%). There were 771 (30.5%) normal weight patients (BMI 18.5–24.9 kg/m^2^) and underweight patients (BMI < 18.5 kg/m^2^) formed the smallest group (*n* = 28, 1.2%).

The age was lowest in the group of underweight patients (56.6 ± 21.7 years) and highest amongst patients in the group of overweight patients (69.4 ± 12.9 years). The distribution of female patients ranged from 22.0% (obesity class I) to 57.1% (underweight). As expected, metabolic comorbidities such as arterial hypertension (84.2%) and diabetes mellitus (51.1%) were highest amongst patients with the highest BMI (obesity class 2; *p* < 0.001). There was a trend towards higher rates of patients with atrial fibrillation at baseline in the obesity groups (23.3% in obesity class 2). Previous cardiac surgery, coronary artery disease or pacemaker dependency were not significantly different between the predefined groups.

### 3.2. Lead and Device Characteristics

Significant differences between the five groups could be seen in the rates of indwelling pacemakers, as well as cardiac resynchronization therapy (CRT) devices, but not in the rates of implantable cardioverter–defibrillators (ICDs). The pacemaker rates were lowest amongst underweight patients (32.1%) and highest amongst normal weight patients (45.1%). CRT devices could be found significantly more frequent in the obesity class 1 and 2 groups at 31.6% and 32.0%, respectively. Indwelling ICD rates ranged between 30.6% (obesity class 1) and 42.9% (underweight patients), but they did not differ significantly. With a total of 16 implanted cardiac contractility management (CCM) devices, numbers were too low to be significantly different between the five patient groups. The age of the oldest indwelling lead varied between a median of 85.5 (55.0; 120.75) months in the highest obesity classes and 99.0 (65.0; 149.0) months in the normal weight group. Right-sided leads could be found predominantly in underweight patients (46.4%), whereas patients with a BMI of 35 kg/m^2^ and above were less likely to be implanted via right-sided approach (27.8%; *p* = 0.029).

The mean number of total leads, as well as the number of abandoned leads, was not different between groups. More detailed information is shown in Table 1.

### 3.3. Procedural Data

Between the predefined patient groups, a trend could be seen in the median hospitalization period, which varied between 8.5 (6.75; 17.25) days (underweight patients) and 10.0 (6; 21) days (obesity class 2). The median postoperative stay ranged between a median of 6 and 7 days.

A trend could be seen towards longer median procedural time in underweight patients (105.5 [58.25; 155.75] days) in comparison to almost similar median procedure times of 79.5 (55.5; 125.0) to 85.0 (60.0; 140.0) days in the other four groups (*p* = 0.450).

Neither minor complications (*n* = 57, *p* = 0.684) nor major complications (*n* = 52, *p* = 0.498) showed significant different numbers in the five predefined BMI groups.

The highest percentage of superior vena cava (SVC) lacerations as the most frequently occurring major complication was seen in the obesity class 2 group (*n* = 3, 2.3%). The distribution of other major complications such as laceration of the right atrium or right ventricle, as well as pericardial tamponade or hemothorax, did not yield significant results.

Clinical procedural success was achieved in 100% (*n* = 28) of underweight patients and rates declined non-significantly towards 95.5% (*n* = 127) in the obesity class 2 group (*p* = 0.504). Complete procedural success ranged between 85.7% (*n* = 24, underweight patients) and 92.0% (*n* = 1082, overweight patients) without being statistically significant (*p* = 0.437) between groups.

Procedure-related death was generally low (*n* = 15), but highest amongst patients with a BMI of 35 kg/m^2^ and above (1.5%), even though it was not significantly different from the other BMI groups (*p* = 0.533).

Of the 90 total fatalities, there was no significant accumulation in one of the groups (*p* = 0.333). Causes of death were evenly distributed and a detailed list can be found together with more data on procedural outcomes in Table 2.

### 3.4. Inter-Group Differences on Patient Characteristics and Procedural Data

Most of the patient groups showed significant differences in relation to the patient’s ages. Patients of obesity class 2 were significantly younger than patients of obesity class 1 (*p* = 0.003), overweight patients (*p* < 0.001) and normal weight patients (*p* < 0.001), but did not differ significantly in age when compared to underweight patients (*p* > 0.99). Patients of obesity class 1 were significantly younger than patients of the overweight group (*p* = 0.04) but did not differ significantly from the normal weight (*p* > 0.99) and underweight groups (*p* = 0.18). Patients of the overweight group and normal weight group also did not differ significantly in age (*p* > 0.99) but were significantly older than patients from the underweight group (*p* = 0.01), and, finally, normal weight patients were significantly older than underweight patients (*p* = 0.04). 

As expected, all weight classes showed significantly different BMI values, but there were no significant inter-group differences in relation to the number of implanted leads, procedural time, lead age, general hospitalization duration and length of postoperative stay. Only patients of the obesity class 1 group had a significant longer preoperative hospitalization period compared to overweight patients (*p* = 0.02), whereas there were no significant inter-group differences between all other patient groups. 

### 3.5. Predictors of Adverse Procedural Outcomes in Obese Patients

To determine possible predictors of adverse events in a composite of patients with obesity class 1–2 (BMI ≥ 30 kg/m^2^), namely procedural failure, complications and all-cause mortality, a univariate regression analysis was performed and the identified predictor candidates were further analyzed via multivariate regression analysis. 

Tested parameters included the existence of abandoned leads, a patient age ≥75 years, the existence of ≥4 leads in situ, systemic infection, coronary artery disease, previous cardiac surgery, a lead age of ≥10 years, a left ventricular ejection fraction (LVEF) of <30%, chronic kidney disease (CKD) and periprocedural complications. 

**Figure 1:**
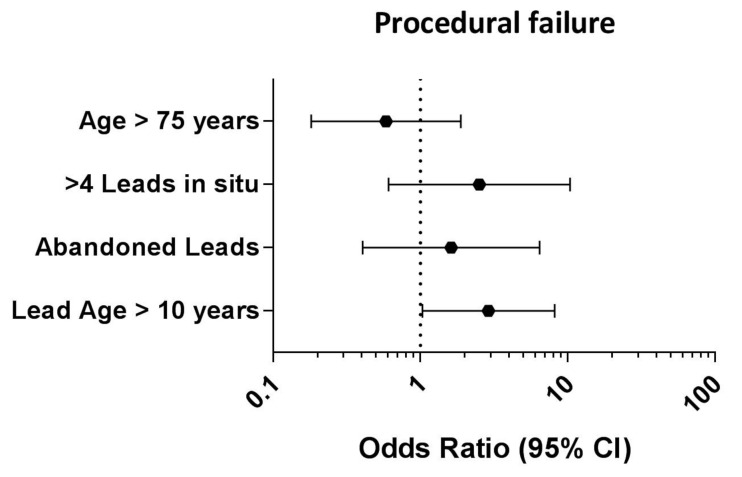
Forest plot depicting the results of the multivariate logistic regression analysis to identify independent predictors for procedural failure in patients with a BMI ≥ 30 kg/m^2^. CI: confidence interval.

As an independent predictor for procedural failure, the lead age ≥10 years (OR: 2.99; 95% CI: 1.06–8.45; *p* = 0.038) was identified as such. All other tested variables did not predict the procedural failure independently. The results of this analysis are depicted via forest plot and are shown in Figure 1.

**Figure 2:**
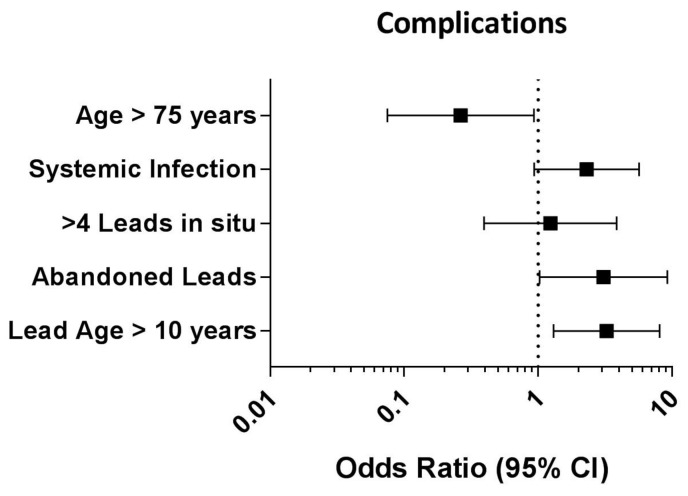
Forest plot depicting the results of the multivariate logistic regression analysis to identify independent predictors for procedural complications in patients with a BMI ≥ 30 kg/m^2^. CI: confidence interval.

Lead age ≥10 years (OR: 3.25; 95% CI: 1.31–8.10; *p* = 0.011) and the existence of abandoned leads (OR: 3.08; 95% CI: 1.03–9.22; *p* = 0.044) were identified as independent predictors for procedural complications, whereas patient age ≥75 years (OR: 0.27; 95% CI: 0.08–0.93; *p* = 0.039) seemed to be associated with a reduced procedural complication rate (see Figure 2).

**Figure 3:**
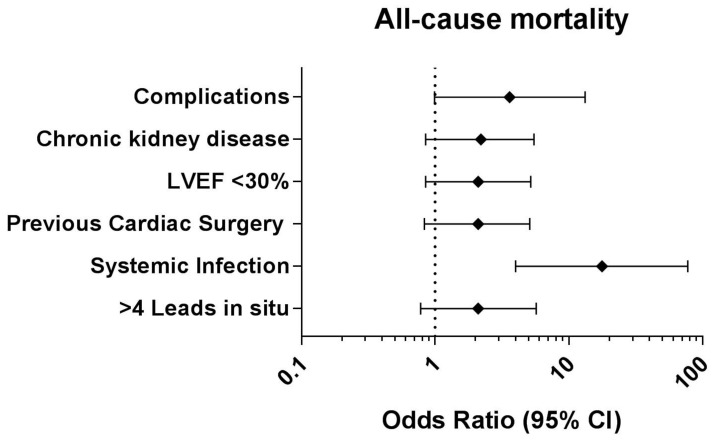
Forest plot depicting the results of the multivariate logistic regression analysis to identify independent predictors for all-cause mortality in patients with a BMI ≥ 30 kg/m^2^. CI: confidence interval.

Finally, periprocedural complications (OR: 5.33; 95% CI: 1.55–18.32; *p* = 0.008) and CKD (OR: 3.20; 95% CI: 1.32–7.80; *p* = 0.010) were identified as independent predictors for all-cause mortality. After adjustment of the model, systemic infection (OR: 17.68; 95% CI: 4.03–77.49; *p* < 0.001) was identified as the sole predictor for all-cause mortality (see Figure 3). 

## 4. Discussion

This study is a subgroup analysis of one of the largest multi-centric patient cohorts undergoing LLE in Germany and focusses on the effects of body weight on patient-centered outcome parameters during and after the lead extraction procedure. The main findings include:

(1) Major (*p* = 0.498) and minor (*p* = 0.684) complication rates in patients receiving LLE were generally low and were not distributed differently between BMI groups.
(2) A lead age ≥10 years and the existence of abandoned leads were independent predictors of complications in obese patients.
(3) Periprocedural complications and CKD could be identified as independent predictors for all-cause mortality. As in other cohorts of GALLERY, after adjustment, systemic infection remained the sole independent predictor of all-cause mortality (17-fold) in obese patients.
(4) Indwelling leads with an age ≥10 years were found as an independent predictor of procedural failure.
(5) A patient age ≥75 years was associated with fewer overall complications in obese patients (obesity classes 1 and 2).
(6) Obesity did not lead to significantly longer procedural or longer postoperative hospitalization time.

### 4.1. Impact of Obesity on Surgical Procedures vs. Minimal Invasive Procedures

Obesity is a major risk factor for the development of metabolic syndrome and plays a substantial role in the development of an inflammatory state that contributes to cardiovascular diseases [2,23].

Interestingly, the impact of obesity on perioperative outcomes seems to be different from its impact on minimal invasive procedures. Obesity led to impaired wound healing and local infections after surgery in a nationwide British study [24], as well as a significantly increased number of deaths and major complications after trauma surgery [13]. Furthermore, a recent investigation found an association of an elevated BMI with increased mortality, higher morbidity and cost for hospital care in patients undergoing cardiothoracic surgery [25].

On the contrary, multiple catheter-based trials did not show increased complications or reduced acute success rates in obese patients [16,26,27].

A possible explanation is a much smaller access site with less potential for wound healing complications and infection. Also, new imaging techniques with higher resolution (point-of-care ultrasound or fluoroscopy) and new devices for vascular closure help operators to work more precisely and cause less trauma to surrounding tissue and vessels.

Furthermore, interventionalists may act with extra care when presented with an obese patient and possibly adjust their procedures accordingly. A possible selection bias towards more “healthy” obese patients cannot be excluded.

### 4.2. Obesity and Procedural Complications/Mortality after LLE

Four lead extraction trials/registries reported periprocedural outcomes over the last three decades [8,10,11,28]. Generally, complication rates and mortality after lead extraction procedures were low.

The ELECTRa study, which gathered information about patients from 73 European centers with lead extraction, showed a major complication rate (including mortality) of 1.7% which is in line with the findings of the GALLERY registry. However, a direct comparison is not possible, since only 19.3% of all procedures were laser-powered and the mean lead-dwelling time, as well as the number of implanted leads, were lower in ELECTRa than in our patient cohort. The mean BMI in ELECTRa was 26.6 ± 4.8 kg/m^2^ which is comparable to the GALLERY patient cohort. In line with the results of our present study, the investigators did not find an elevated BMI to be an independent predictor of mortality [8,28]. Consequently, a proposed risk score (EROS score) for adverse events after TLE, validated in the ELECTRa cohort, did not include the BMI in its calculation, but a possible impact of severe underweight or obesity has not been studied [19].

In line with the earlier mentioned retrospective single-center study [17], a patient registry from 2010, the LExICon trial, looked for predictors of periprocedural complications and death after LLE, and identified a BMI ≤ 25 kg/m^2^ as a risk factor amongst others, whereas a higher BMI (>25 kg/m^2^) did not predict adverse events [11]. The investigators did not stratify the obese patients, so a more detailed analysis on the impact of obesity on periprocedural complications and death was not possible. In our trial, we did not see such an impact of patients with normal and low BMI on procedural outcomes of the LLE. It must be mentioned, though, that the number of patients in the underweight group was too small to further analyze its impact on adverse events.

In the present analysis we did not observe a significant increase of complication rates in obese patient groups, and generally low complication rates of 2.1% for major complications and 2.3% for minor complications imply the safety of LLE amongst all patient groups.

### 4.3. Systemic Infection and Obesity in LLE Procedures

The analyses of possible predictors for all-cause mortality after LLE procedures revealed systemic infection as sole, independent predictor in patients with a BMI of ≥30 kg/m^2^. This effect is in line with another subgroup analysis of the GALLERY registry, which found systemic infection (cardiac-device-related systemic infection) to be a major risk factor for periprocedural and all-cause mortality after LLE procedure, despite comparable procedural success rates with patients presenting without systemic infection [9]. In the present analysis, most patients with a BMI of ≥35 kg/m^2^ needed LLE because of systemic infection which differed significantly from normal weight patients. Obesity has been shown to be an independent predictor for systemic infection [9], as well as a risk factor (abdominal obesity, in addition to underweight patients) for infection-related mortality in a general, non-CIED-related population [29]; thus, it seems paramount to implement strategies for weight reduction, beginning with education and coaching of patients, especially with indwelling CIEDs.

### 4.4. Significance of Age in Relation to Procedural Complications in Obese Patients

Interestingly, the results of our multivariate regression analysis suggested a protective effect of a patient age ≥75 years for obese patients in relation to acute complications in LLE procedures. This seemed to be counterintuitive at first but is in line with a recent study from Yayanama et al. who found that a BMI of 30.0–34.9 kg/m^2^ was protective for mortality in patients with a higher frailty index. At the same time, a BMI of 35.0 kg/m^2^ and above was detrimental, especially in patients with lower frailty indices [30]. In another study on the influence of overweight on frailty in elderly patients, the authors discussed a potential benefit of a higher BMI in this patient group that normally shows a negative energy balance and is prone to malnutrition and frailty [31].

This supports the general idea of an obesity paradox, that has been discussed in surgical trials previously [14,15]. Although obesity has been shown to be a major risk factor for several cardiovascular diseases [2], it seems to mediate the negative impact of frailty and emaciation amongst the elderly. Definitive evidence on the obesity paradox is hard to come by, especially since the BMI delivers a measurement uncertainty in relation to obesity in this discussion. Amongst others, body fat, muscle mass and bone structure are included in the parameter and should be analyzed separately, but, as stated in our limitations, this is a well-known detriment of this surrogate marker.

Furthermore, another factor that may come into play seems to be the generally higher rates of calcification of the adhesive thrombo-fibrotic lead encapsulations of implanted leads in younger patients, as well as patients with longer lead-dwelling times [32]. Calcification may lead to a higher rate of laser-powered extraction devices and risks of perforation, especially in the area of the superior vena cava, are high [33].

It should be mentioned that our data reflects a certain reciprocal effect of obesity, since underweight showed a high risk for all-cause mortality, complications and procedural failure in octo- and nonagenarians undergoing lead extraction (Pecha et al., currently under review).

### 4.5. Limitations

This study is a retrospective, subgroup analysis of a large registry; thus, the possibility of detection and selection bias is given.

With only 28 patients in the underweight patient group, numbers were generally too low to adequately analyze and identify the influence of underweight on the different measured patient outcomes.

As a surrogate parameter for obesity, the BMI has its obvious and documented limitations, although it is widely accepted as a screening tool [34]. A measurement of body fat or waist circumference could possibly have altered the results of our study to some extent but, as an easily acquired parameter (and with better comparability), the BMI was used in this analysis. It also generally correlates well with health outcomes in population-based trials.

Finally, only high-volume centers with at least 20 LLE procedures per year participated in the GALLERY registry, so a generalization to centers with less experience is not possible.

## 5. Conclusions

With considerate planning and in experienced centers, LLE is a generally safe and efficacious extraction option for obese patients. Complication rates and (periprocedural) mortality for obese patients did not differ from normal weight patients, and procedural time as well as duration of hospitalization was not prolonged. Systemic infection is the main driver of mortality in patients with a BMI ≥ 30 kg/m^2^.

## Figures and Tables

**Table 1:** Patient and device characteristics.

	BMI < 18.5 kg/m^2^ (*n* = 28)	BMI ≥ 18.5–24.9 kg/m^2^ (*n* = 771)	BMI ≥ 25–29.9 kg/m^2^ (*n* = 1176)	BMI ≥ 30–34.9 kg/m^2^ (*n* = 422)	BMI ≥ 35 kg/m^2^ (*n* = 133)	*p* Value
**Mean age, years** **± SD**	56.6 ± 21.7	67.5 ± 15.7	69.4 ± 12.9	67.7 ± 11.9	63.4 ± 11.2	<0.001
**Female sex, *n* (%)**	16 (57.1)	234 (30.4)	558 (47.4)	93 (22.0)	41 (30.8)	<0.001
**Mean BMI, kg/m^2^ ± SD**	17.4 ± 0.68	22.6 ± 1.62	27.2 ± 1.23	31.9 ± 1.36	39.1 ± 4.17	<0.001
**LVEF ≤ 30%, *n* (%)**	6 (21.4)	181 (23.5)	301 (25.6)	127 (30.1)	42 (31.5)	0.067
**Arterial hypertension, *n* (%)**	11 (39.8)	476 (61.7)	850 (72.3)	330 (78.2)	112 (84.2)	<0.001
**Coronary artery disease, *n* (%)**	6 (21.4)	319 (41.4)	512 (43.5)	189 (44.8)	57 (42.9)	0.147
**Diabetes mellitus, *n* (%)**	5 (17.6)	181 (23.5)	345 (29.3)	190 (45.0)	68 (51.1)	<0.001
**Chronic kidney disease, *n* (%)**	8 (28.6)	218 (28.3)	355 (30.2)	155 (36.7)	49 (36.8)	0.020
**Previous heart surgery, *n* (%)**	4 (14.3)	183 (23.7)	285 (24.2)	112 (26.5)	28 (21.1)	0.467
**Pacemaker dependency, *n* (%)**	7 (25.0)	265 (34.4)	371 (31.5)	123 (29.1)	37 (27.8)	0.256
**ECG at admission**						
- **Sinus rhythm, *n* (%)**	18 (64.3)	392 (50.8)	601 (51.1)	227 (53.8)	65 (48.9)	0.510
- **Atrial fibrillation, *n* (%)**	5 (17.6)	146 (18.9)	242 (20.6)	86 (20.4)	31 (23.3)	0.778
- **Paced rhythm, *n* (%)**	5 (17.6)	233 (30.2)	327 (27.8)	109 (25.8)	37 (27.8)	0.367
**Extraction indication**						
**Local infection, *n* (%)**	10 (35.7)	257 (33.3)	441 (37.5)	147 (34.8)	33 (24.8)	0.071
**Systemic infection, *n* (%)**	4 (14.3)	229 (29.7)	297 (25.3)	139 (32.9)	53 (39.8)	<0.001
**Lead dysfunction, *n* (%)**	11 (39.8)	239 (31.0)	370 (31.5)	112 (26.9)	44 (33.1)	0.276
**Device upgrade, *n* (%)**	0 (0)	16 (2.1)	29 (2.5)	11 (2.6)	0 (0)	0.359
**Vascular complication, *n* (%)**	1 (3.6)	6 (0.8)	7 (0.6)	3 (0.7)	0 (0)	0.323
**Chronic pain, *n* (%)**	0 (0)	8 (1.0)	7 (0.6)	1 (0.2)	0 (0)	0.391
**Other, *n* (%)**	2 (7.1)	16 (2.1)	19 (1.6)	9 (2.1)	3 (2.3)	0.301
**Device and lead characteristics**						
**Pacemaker, *n* (%)**	9 (32.1)	348 (45.1)	473 (40.2)	159 (37.7)	40 (30.1)	0.004
**ICD, *n* (%)**	12 (42.9)	253 (32.8)	411 (34.9)	129 (30.6)	50 (37.6)	0.300
**CRT, *n* (%)**	7 (25.0)	172 (22.3)	287 (24.4)	135 (32.0)	42 (31.6)	0.002
**CCM, *n* (%)**	0 (0)	1 (0.1)	11 (0.9)	2 (0.5)	2 (1.5)	0.148
**Total number of leads, *n***	67	1862	2820	1051	317	
**Mean number of total leads, *n*** **± SD**	2.4 ± 1.0	2.4 ± 1.0	2.4 ± 1.0	2.5 ± 1.0	2.4 ± 1.0	0.500
**Median age of oldest lead, months [IQR]**	90.5	99.0	96.0	93.0	85.5	0.05
	[55; 126.75]	[65; 149]	[62; 140]	[61; 138.5]	[55; 120.75]	
**Number of patients with right sided leads, *n* (%)**	13 (46.4)	282 (36.6)	384 (32.7)	124 (29.4)	37 (27.8)	0.029
**Number of patients with abandoned leads, *n* (%)**	9 (32.1)	246 (31.9)	338 (28.7)	139 (32.9)	28 (21.1)	0.061

Values are expressed as mean ± SD, or counts (*n*) and percentages (%); χ^2^-test was used for categorical variables; Kruskal–Wallis test was used for continuous variables; *p*-values < 0.05 were considered statistically significant; BMI: body mass index; CCM: cardiac contractility modulation; CRT: cardiac resynchronization therapy; ECG: electrocardiogram; ICD: implantable cardioverter–defibrillator; IQR: interquartile range; LVEF: left ventricular ejection fraction; SD: standard deviation.

**Table 2:** Procedural outcomes.

	BMI < 18.5 kg/m^2^ (*n* = 28)	BMI ≥ 18.5–24.9 kg/m^2^ (*n* = 771)	BMI ≥ 25–29.9 kg/m^2^ (*n* = 1176)	BMI ≥ 30–34.9 kg/m^2^ (*n* = 422)	BMI ≥ 35 kg/m^2^ (*n* = 133)	*p* Value
**Median hospital stay, days [IQR]**	8.5	9.0	9.0	10.0	10.0	0.010
	[6.75; 17.25]	[6; 16]	[5;15]	[6; 18]	[6; 21]	
**Median duration from admission to procedure, days [IQR]**	1.0 [1; 3]	2.0 [1; 4]	2.0 [1; 3.14]	2.0 [1;4]	2.0 [1; 4]	0.020
**Median postoperative stay, days [IQR]**	7.0 [5; 14.25]	6.0 [3; 13]	6.0 [3; 12]	6.8 [4; 12]	7.0 [3; 15]	0.09
**Median procedural time, minutes [IQR]**	105.5 [58.25; 155.75]	81.0	81.0	79.5	85.0	0.450
		[57; 130]	[55; 121.75]	[55.5; 125]	[60; 140]	
**Overall complications, *n* (%)**	2 (7.1)	42 (5.4)	43 (3.7)	17 (4.0)	5 (3.8)	0.359
**Minor complications, *n* (%)**	1 (3.6)	22 (2.9)	24 (2.0)	8 (1.9)	2 (1.5)	0.684
**Pocket hematoma, *n* (%)**	-	18 (2.3)	22 (1.9)	5 (1.2)	2 (1.5)	
**Pericardial effusion without intervention, *n* (%)**	1 (3.6)	1 (0.1)	-	2 (0.5)	-	
**Pneumothorax, *n* (%)**	-	2 (0.3)	2 (0.2)	-	-	
**Subclavian vein thrombosis, *n* (%)**	-	-	-	1 (0.2)	-	
**Pulmonary embolism, *n* (%)**	-	1 (0.1)	-	-	-	
**Major complications, *n* (%)**	1 (3.6)	20 (2.6)	19 (1.6)	9 (2.1)	3 (2.3)	0.498
**Laceration of SVC or cavo-atrial junction, *n* (%)**	-	10 (1.3)	8 (0.7)	1 (0.2)	3 (2.3)	
**RA/RV perforation, *n* (%)**	-	3 (0.4)	10 (0.9)	6 (1.4)	-	
**Pericardial tamponade, *n* (%)**	1 (3.6)	4 (0.5)	1 (0.1)	2 (0.5)	-	
**Hemothorax, *n* (%)**	-	3 (0.4)	-	-	-	
**Procedural outcomes**						
**Complete procedural success, *n* (%)**	24 (85.7)	697 (90.4)	1082 (92.0)	387 (91.7)	118 (88.7)	0.437
**Clinical procedural success, *n* (%)**	28 (100.0)	754 (97.8)	1148 (97.6)	412 (97.6)	127 (95.5)	0.504
**Procedure-related mortality, *n* (%)**	0 (0)	5 (0.6)	5 (0.4)	2 (0.5)	2 (1.5)	0.580
**All-cause mortality, *n* (%)**	2 (7.1)	31 (4.0)	33 (2.8)	19 (4.5)	5 (3.8)	0.333
**Causes of death**						
**Septic shock, *n* (%)**	-	19 (2.5)	19 (1.6)	11 (2.6)	2 (1.5)	
**Multiorgan failure (not further specified), *n* (%)**	-	5 (0.6)	5 (0.4)	5 (1.2)	2 (1.5)	
**Cardiogenic shock, *n* (%)**	1 (3.6)	3 (0.4)	2 (0.2)	3 (0.7)	-	
**Hemorrhagic shock, *n* (%)**	-	2 (0.3)	1 (0.1)	-	-	
**Mesenteric ischemia, *n* (%)**	-	-	2 (0.2)	-	1 (0.8)	
**Fatal arrhythmia (VT/VF), *n* (%)**	1 (3.6)	-	1 (0.1)	-	-	
**Asystole, *n* (%)**	-	-	1 (0.1)	-	-	
**Pericardial tamponade, *n* (%)**	-	1 (0.1)	-	-	-	
**Fatal pulmonary embolism, *n* (%)**	-	-	1 (0.1)	-	-	
**Cerebrovascular accident, *n* (%)**	-	1 (0.1)	-	-	-	
**Acute renal failure, *n* (%)**	-	-	1 (0.1)	-	-	

Values are expressed as mean ± SD, or counts (*n*) and percentages (%); χ^2^-test was used for categorical variables; Kruskal–Wallis test was used for continuous variables; *p*-values < 0.05 were considered statistically significant; BMI: body mass index; IQR: interquartile range; RA: rights atrium; RV: right ventricle; SVC: superior vena cava.

## Data Availability

No new data were created or analyzed in this study. Data sharing is not applicable to this article. For original data see citation 8 (GALLERY).
